# Do discharge delays explain longer stays at veterans health administration hospitals?

**DOI:** 10.1186/s12913-025-13682-w

**Published:** 2025-12-12

**Authors:** Brian P. Lucas, Daniel M. Weinberger, Anita A. Vashi, Jeremy Smith, Caroline Korves, Amy C. Justice, Britta I. Neugaard, Louise Davies

**Affiliations:** 1 VA Outcomes Group, Department of Veterans Affairs Medical Center, White River Junction, Vermont, USA; 2 The Dartmouth Institute of Health Policy & Clinical Practice, Geisel School of Medicine at Dartmouth, Hanover, New Hampshire USA; 3 Yale Schools of Public Health, New Haven, Connecticut USA; 4 Department of Veterans Affairs Connecticut Healthcare System, West Haven, Connecticut USA; 5 Center for Innovation to Implementation, Veterans Affairs Palo Alto Health Care System, Menlo Park, California USA; 6 Department of Emergency Medicine, University of California, San Francisco, California USA; 7 White River Junction VA Medical Center, White River Junction, Vermont USA; 8 Yale Schools of Medicine and Public Health, New Haven, CT USA; 9 Office of Utilization Management, Veterans Health Affairs National Patient Safety Center, Washington, DC USA; 10 Department of Surgery-Otolaryngology Head & Neck Surgery, Geisel School of Medicine at Dartmouth, Hanover, New Hampshire USA

**Keywords:** Length of stay, Hospitalization, Patient discharge, Patient transfer, Aftercare, Continuity of patient care

## Abstract

**Background:**

Discharge delays are difficult to quantify without standardized indicators for when patients are medically ready for discharge. We aimed to estimate the proportion of increased hospital length of stay attributable to discharge delays, as proxied by increases in ‘avoidable’ days.

**Methods:**

We conducted a retrospective cohort study of Veterans Health Administration hospitals in the continental United States with emergency departments between 1 March 2019 and 28 February 2023. We included Veterans who were discharged from an acute medicine service without a COVID-19 diagnosis. We used standardized utilization management criteria to count ‘avoidable’ days, defined as hospital days when acute care was no longer required. Our primary outcome was geometric mean length of the discharging stay (the final acute medicine segment prior to discharge), which reflects the time most susceptible to discharge delays.

**Results:**

During the study period there were 868,031 eligible hospitalizations. Adjusted geometric mean length of discharging stay increased 9.3% (95% CI, 8.7% to 9.9%) from the pre-pandemic year to the third pandemic year, with the largest increase among discharges to facility-based post-acute care (23.3% [95% CI, 21.6 to 24.9%]). However, among all hospitalizations only 16% (95% CI, 15 to 17%) of the increase in discharging stay was attributable to an increase in avoidable days.

**Conclusions:**

Most of the increase in length of hospital discharging stay was not explained by discharge delays and may instead reflect longer periods of acute care delivery. Improving acute care processes may more effectively reduce hospital capacity strain than bolstering post-acute care availability.

**Supplementary Information:**

The online version contains supplementary material available at 10.1186/s12913-025-13682-w.

## Introduction

Shortages of hospital beds—exacerbated by the onset of the COVID-19 pandemic [1]—have renewed attention to discharge delays as targets for improving inpatient capacity [2, 3]. Yet the impacts of discharge delays are difficult to quantify [4]. Most electronic medical records lack standardized indicators for when acute care delivery is complete or patients are “medically ready for discharge” [5]. How much reductions in discharge delays would improve hospital capacity strain (or “gridlock”) [6] is not well known [7].

To address this gap, we leveraged standardized utilization review data from Veterans Health Administration (VHA) hospitals to assess whether rising discharge delays contributed to longer hospital stays. Specifically, we examined trends in “avoidable” days—hospital days for which, according to utilization reviewers, acute-level care was no longer needed. We tested whether the rise in “avoidable” days, as a proxy for the rise in discharge delays, could explain the observed increase in length of discharging stay, the final stay on the acute medicine service before discharge and the portion of hospitalization most sensitive to discharge delays.

## Methods

### Cohort creation

After the Veteran’s Institutional Reviewer Board of Northern New England granted exemption from informed consent (VINNE 1,641,396), we used the methods of Vincent et al. To establish start and stop times for hospitalizations within the VHA Corporate Data Warehouse (CDW) [8]. Patients’ movements within and across VHA facilities are indexed as “bedded stays”—durations of time when a patient is provided a bed at a particular location. A bedded stay may be a complete stand-alone hospitalization or just part of one. As patients move bed locations, multiple bedded stays combine to form hospitalizations [9]. In addition, bedded stays themselves may be comprised of shorter specialty stays: unique combinations of locations and specialties (also known as services or specialty services). We delineated hospitalizations by first extracting the timestamps to split all bedded stays into specialty stays (Additional File 1). We then removed Extended Nursing or Housing specialty stays, as defined in Additional File 2, and spliced together adjacent specialty stays. We developed rules to deal with overlapping stays, which sometimes occur when a patient administratively occupies more than one bed at a time. We validated our method by comparing weekly national medicine and surgery inpatient census totals against a wholly separate database that is manually compiled by bed flow coordinators at all VHA hospitals. As described in Additional File 3, the census totals from both methods were remarkably similar across the study period, validating our approach.

**Fig. 1:**
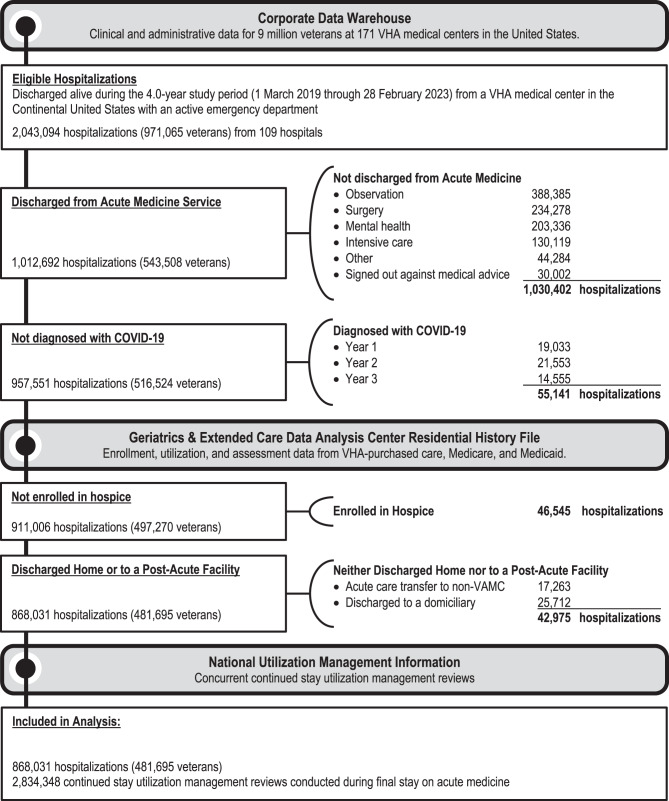
Cohort creation and data linkages. Abbreviations: VHA = veterans health administration affairs medical center; COVID-19 = coronavirus disease 2019. For the definitions of criteria used in this figure, see Additional file 4

We identified eligible hospitalizations of VHA enrollees at one of 109 VHA hospitals in the continental US with an emergency department throughout the 4-year study period—from 1 year before to 3 years after February 29, 2020, the date of the first COVID-19 related death in the US (Fig. 1). We restricted hospitalizations to those in which patients were discharged from an acute medicine service, and we excluded hospitalizations in which patients signed out against medical advice or had a COVID-19 diagnosis.

We then linked to the Geriatrics & Extended Care Data Analysis Center Residential History File, which combines enrollment, utilization, and assessment data from VHA-care, VHA-purchased care, Medicare, and Medicaid to determine veteran movements between health care settings [10].

We used this file to exclude veterans who were enrolled in hospice, transferred to a non-VHA hospital for ongoing acute care, or discharged to a domiciliary. Further details about the definitions used to define our cohort are listed in Additional File 4.

### Utilization review data

At VHA hospitals, trained nurses conduct concurrent utilization management reviews to determine if hospitalized patients meet standardized InterQual^®^ criteria for continued stay at an acute level of care [11]. Utilization management reviewers are expected to conduct continued stay reviews for at least 80% of bed-days (1 hospital bed occupied for 1 day comprises 1 bed-day unit; we use the terms “bed-day” and “day” interchangeably); this expectation does not include days of hospital admission (which are evaluated against admission criteria), days of discharge, days beyond 30-days on the same specialty, or days enrolled in hospice care.

We retrieved archived reviews from the National Utilization Management Integration (NUMI) database and included any that were conducted during a patient’s final acute medicine stay. Given that such reviews are not conducted daily, we generated 2 rules to impute identical reviews across bed days within hospitalizations. First, we interpolated reviews when they were identical for bed-days before and after a gap. Second, we carried forward the last review that was conducted during a hospitalization if, during a 7-day period that ended with that last review, all of 2 or more reviews were identical. A day was classified as “avoidable” if a reviewer determined that 1) the patient no longer met continued stay criteria and 2) care could be provided at a lower level of acuity, such as at home or in a skilled nursing facility [12].

Continued stay criteria were based on disease-specific InterQual® criteria, which the VHA has been using to conduct utilization management reviews since 1993. Generally, patients meet criteria for continued stay when interventions are still needed for continued clinical improvement or for addressing barriers to a safe discharge. For example, a patient with severe pneumonia who, on hospital day 4, no longer requires intravenous antibiotics or supplemental oxygen, tolerates oral medication, breathes comfortably, and maintains stable vital signs, may nevertheless meet continued stay criteria if he continues to lack functional independence. If barriers to a safe discharge are then addressed on day 7, for example, by the patient regaining functional independence , he would no longer meet continued stay criteria. And if reviewers also determined that care could be provided at a lower level of acuity, then day 7 and any additional days until hospital discharge would meet our definition of avoidable.

### Statistical analysis

Our primary outcome was the length of discharging stay, defined as the number of days on the final acute medicine service before discharge; we transformed the length of discharging stay (measured in days) to the natural log scale (log_e_(days)) to reduce the influence of extreme values and improve model convergence. We chose this segment of hospitalization to focus on the impact of delays related to hospital discharge, rather than the impacts of other factors such as transfers from intensive care or between other acute services [13]. Our exposures of interest were 1) year of discharge, 2) discharge destination (home, at-home post-acute care, or facility-based post-acute care), and 3) avoidable days. Potential confounders were defined at 3 levels: hospital-level were geographic region, rurality, and whether a VHA nursing home (a Community Living Center [CLC]) existed on campus (Additional File 5); patient-level were sex, race and ethnicity; and hospitalization-level were age at discharge, comorbidity at discharge, pre-hospital care, admission from a non-VHA hospital, admission to observation status, stays on specialties other than acute medicine, post-acute facility type, and similarity of pre- and post-hospital care. Further details about how exposures of interest and potential confounders were defined are listed in Additional File 6.

Patients are often hospitalized more than once, but they do not always return to the same hospital. To correctly adjust for the clustering of unique combinations of patients and hospitals, we built a nonhierarchical mixed effects model with additive crossed random effects for both patients and hospitals using a maximum likelihood estimator with the mixed command in Stata 18.0. For fixed effects, we entered discharge destination (an exposure of interest) into our model as both a main effect and as an interaction with year to allow its impact to vary by year. We entered avoidable bed days (another exposure of interest) and all potential confounders as both main effects and as interactions with year and discharge destination singly (as two-way interactions) and combined (as three-way interactions) to allow their impacts on the outcome (discharging length of stay) to vary by year, discharge destination, and the combination of year-by-discharge destination. While inclusion of three-way interactions makes our model less parsimonious, our aim was to capture the full complexity of the underlying phenomenon. Moreover, we present predictions from the entire model, not stand-alone estimates of individual coefficients. And so, the challenge of interpreting three-way interactions was not a drawback.

We used likelihood ratio tests, Akaike’s information criterion, and Bayesian information criterion to assess our fully ‘saturated’ model (with two- and three-way interactions, as described above) against simpler, less saturated versions. In all cases, models saturated with more interactions had improved fit over less saturated models, and so we retained the fully saturated model. Finally, we assessed the normality assumption of both random effects, as described in Additional File 7.

To assess the impact of avoidable days on discharging length of stay, we used the margins command [14] in Stata 18.0 to generate two sets of model-based predictions (‘conditional margins’) [15], while holding all confounders at their pre-pandemic values. In the first set, avoidable days were held constant at their pre-pandemic year average; in the second, they were allowed to vary by discharge year.

One benefit of log-transforming our outcome variable (length of discharging stay) is clear interpretation of predictions. The arithmetic mean of a log-transformed variable is, upon back-transformation, equal to the geometric mean of the variable in the original scale. And so, because our model predictions are arithmetic means in the natural log scale (log_e_(days)), back-transformation creates *geometric means* of our outcome variable, length of discharging stay. In addition, because length of stay variables often approximate log-normal distributions [16], a geometric mean will approximate a median. Another benefit is that differences between our predictions (arithmetic means in the natural log scale) will equal ratios of geometric means upon back-transformation [17]. These ratios are the relative (or ‘fractional’) changes in geometric mean length of discharging stay (*eg*, the relative increase between pandemic year 3 and the pre-pandemic year). We used the margins command with the contrast option to generate these differences.

Finally, we used the nlcom command in Stata 18.0 to calculate the proportion of the increase in adjusted length of discharging stay attributable to avoidable days by comparing two sets of predictions in which confounders were held at pre-pandemic values. In the first set, avoidable days were also held at pre-pandemic values; in the second, they were allowed to vary by discharge year. The proportion of the increase in adjusted length of discharging stay attributable to avoidable days was calculated by comparing these two sets of predictions for each pandemic year relative to baseline. Both the margins and nlcom commands in Stata 18.0 use the delta method to estimate confidence intervals, which were calculated based on the standard normal distribution.

## Results

Our cohort included 868,031 hospitalizations among 481,695 unique veterans, most of whom were elderly, non-Hispanic white men (Table 1). Annual hospitalizations were 47,703 lower during the pandemic years in comparison to the pre-pandemic year (Table 2); exclusion of veterans with COVID-19 explained less than half of that difference (Fig. 1). Discharge destinations were home (70.1%), at-home post-acute care (16.7%), and facility-based post-acute care (13.2%). In 77.6% of cases, the discharge destination matched the patient’s pre-hospital care setting.

**Table 1:** Characteristics of hospitalizations and veterans in study cohort

	Hospitalizations			
	Overall (n = 868,031)	Discharge Destination		
Characteristic		Home (n = 608,078)	Home with post-acute care (n = 145,367)	**Post-acute facility**^**a**^ (n = 114,586)
Unique patients^b^	481,695	382,595	98,897	82,453
Age, mean (SD), y	70 (13)	68 (13)	74 (11)	76 (10)
Men, %	94.5	94.0	95.6	96.4
Race, %				
White	71.4	70.6	72.6	73.8
Black	25.4	25.8	24.9	23.9
Other^c^	2.1	2.2	1.9	1.8
Unknown	1.1	1.4	0.5	0.5
Hispanic, %	5.1	5.4	4.9	4.1
Geographic region, %^d^				
Northeast	12.8	12.4	12.0	15.6
Midwest	22.7	23.0	20.3	23.9
South	42.9	42.9	45.4	39.5
West	21.7	21.7	22.3	20.9
Elixhauser Mortality Index, median (IQR)	9 (−2 to 22)	7 (−3 to 19)	13 (1 to 27)	15 (2 to 29)
Transferred from a non-VA hospital, %	3.0	2.5	3.3	5.3
Initially admitted as observation status, %	16.0	16.0	17.2	14.5
Previous stay, %				
Intensive care	15.7	14.3	16.7	21.6
Acute mental health	0.39	0.43	0.19	0.42
Acute surgery	1.2	0.8	1.5	2.7
Any intensive care, acute mental health, or acute surgery	16.7	15.2	17.8	23.5
Rural hospital^e^	2.7	2.4	3.4	3.0
VHA nursing home on campus^f^	70.3	70.3	69.1	71.7
Pre-hospital care^g^				
Home	85.8	97.1	59.1	60.0
Home with post-acute care	9.7	2.1	39.5	12.5
Post-acute facility	4.5	0.8	1.4	27.5

Abbreviations: IQR = interquartile range; SD = standard deviation

^a^ Of 114,586 hospitalizations discharged to nursing homes, 41.2% were to VHA nursing homes (Community Living Centers), 40.3% were to skilled nursing facilities, 13.6% were to long-term care facilities (also known as contract nursing homes), and 4.9%% were to State Veterans Homes

^b^ Of 481,695 overall unique patients, 63,968 (13.3%) were discharged to 2 different destinations throughout the study period, and 9,141 (1.9%) were discharged to all 3

^c^ The ‘other’ race category included American Indian, Alaska Native, Asian, Native Hawaiian, and other Pacific Islander

^d^ Geographic region defined by the Centers for Disease Control

^e^ Based on rurality classification of discharging VHA hospital (Additional File 5)

^f^ VHA nursing homes are also known as Community Living Centers (Additional File 5)

^g^ Of the entire cohort, 77.6% (673,875 hospitalizations) had the same types of pre-hospital care and discharge destination, including the same type of post-acute care facility. Among the 31,531 hospitalizations in which veterans were admitted from and discharged to a post-acute facility, 5,333 were discharged to of post-acute care facility that was different from the type on admission

**Table 2:** Length of stay by discharge destination

Year	Annual Hospitalizations		Length of Stay (days)			
	(no.)	Change	Discharging Stay		Hospitalization	
			geometric mean (IQR)	arithmetic mean (SD)	geometric mean (IQR)	arithmetic mean (SD)
**Discharged to Post-Acute Facility**						
Pre-Pandemic Year	35,307	—	5.4 (3.2 to 8.8)	7.6 (10.4)	6.7 (4.0 to 10.8)	9.4 (15.2)
Pandemic Years 1–3	26,426	−25.2%	6.4 (3.9 to 10.9)	9.4 (14.3)	7.9 (4.8 to 13.1)	11.4 (17.4)
Pandemic Year 1	24,159	−31.6%	6.1 (3.7 to 10.7)	9.0 (14.4)	7.6 (4.6 to 12.9)	11.0 (18.8)
Pandemic Year 2	28,497	−19.3%	6.3 (3.9 to 10.7)	9.1 (13.8)	7.8 (4.8 to 12.8)	11.0 (16.7)
Pandemic Year 3	26,623	−24.6%	6.8 (4.0 to 11.7)	10.1 (14.6)	8.4 (5.0 to 13.8)	12.0 (16.8)
**Discharge to Home with At-Home Post-Acute Care**						
Pre-Pandemic Year	35,369	—	3.3 (1.9 to 5.8)	4.6 (5.8)	4.2 (2.7 to 6.8)	5.6 (6.6)
Pandemic Years 1–3	36,666	+3.7%	3.6 (2.1 to 6.3)	5.2 (7.0)	4.6 (2.8 to 7.5)	6.3 (7.8)
Pandemic Year 1	33,992	−3.9%	3.6 (2.0 to 6.1)	5.1 (7.9)	4.5 (2.8 to 7.1)	6.2 (8.6)
Pandemic Year 2	38,643	+9.3%	3.6 (2.1 to 6.3)	5.1 (5.9)	4.6 (2.8 to 7.4)	6.2 (7.0)
Pandemic Year 3	37,363	+5.6%	3.7 (2.1 to 6.7)	5.4 (7.3)	4.7 (2.8 to 7.7)	6.5 (8.0)
**Discharged Home**						
Pre-Pandemic Year	182,109	—	2.5 (1.6 to 4.3)	3.7 (5.4)	3.2 (2.0 to 5.0)	4.5 (6.8)
Pandemic Years 1–3	141,990	−22.0%	2.7 (1.7 to 4.7)	4.1 (7.3)	3.4 (2.0 to 5.6)	4.9 (9.6)
Pandemic Year 1	136,988	−24.8%	2.6 (1.6 to 4.3)	3.9 (7.9)	3.3 (2.0 to 5.4)	4.8 (9.7)
Pandemic Year 2	146,046	−19.8%	2.7 (1.6 to 4.7)	4.0 (6.3)	3.4 (2.0 to 5.5)	4.8 (8.0)
Pandemic Year 3	142,935	−21.5%	2.8 (1.7 to 4.8)	4.3 (7.8)	3.5 (2.0 to 5.7)	5.1 (10.8)
**All Hospitalizations**						
Pre-Pandemic Year	252,785	—	2.9 (1.8 to 5.1)	4.4 (6.5)	3.7 (2.2 to 6.0)	5.3 (8.7)
Pandemic Years 1–3	205,082	−18.9%	3.2 (1.8 to 5.8)	5.0 (8.7)	4.0 (2.4 to 6.8)	6.0 (10.9)
Pandemic Year 1	195,139	−22.8%	3.1 (1.8 to 5.6)	4.7 (9.1)	3.9 (2.3 to 6.6)	5.8 (11.3)
Pandemic Year 2	213,186	−15.7%	3.2 (1.8 to 5.8)	4.9 (7.9)	4.0 (2.4 to 6.7)	5.9 (9.7)
Pandemic Year 3	206,921	−18.1%	3.3 (1.9 to 5.9)	5.2 (9.1)	4.1 (2.5 to 6.9)	6.2 (11.6)

Abbreviations: IQR = interquartile range; SD = standard deviation

**Fig. 2:**
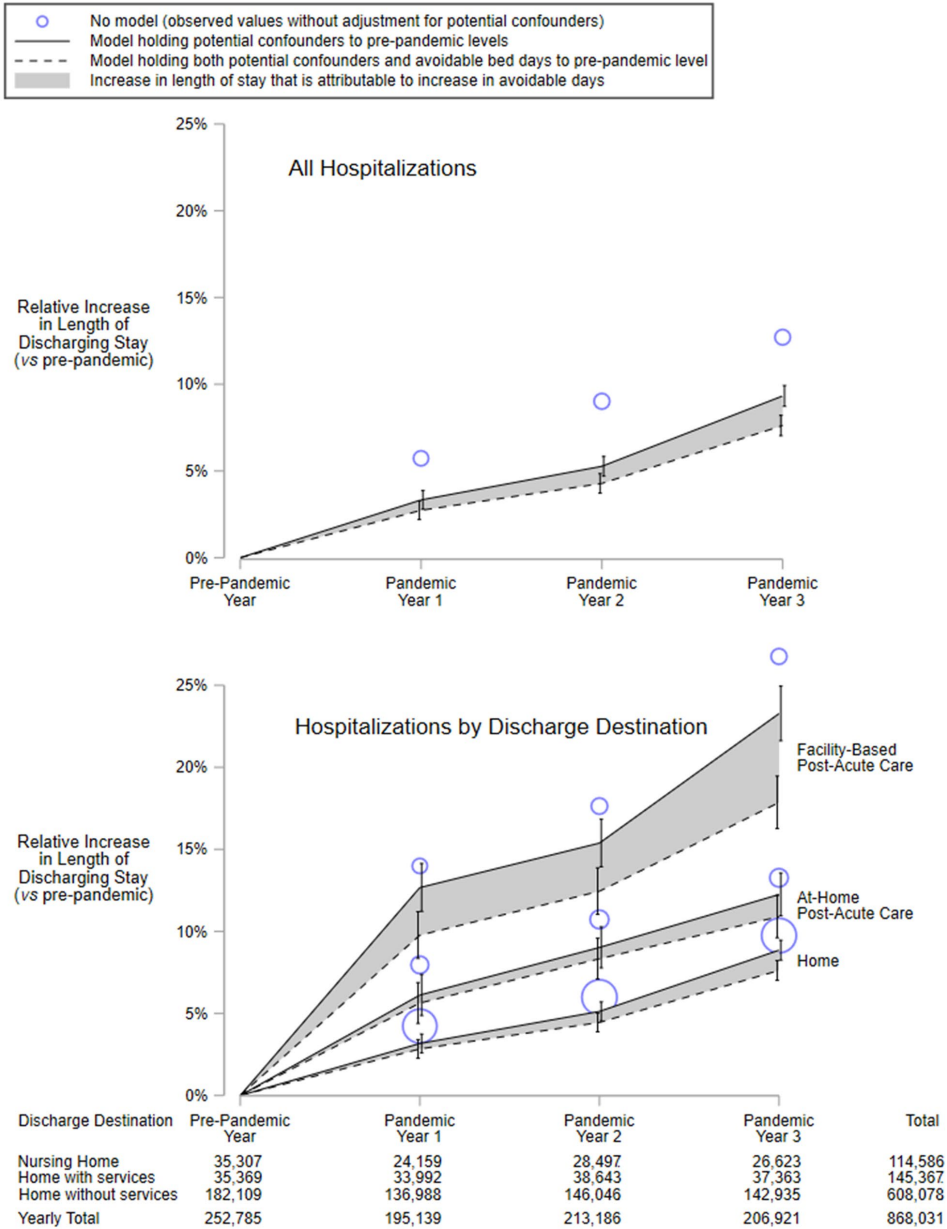
Relative increase in geometric mean length of discharging stay. Both panels depict the relative increase in geometric mean length of discharging stay for each pandemic year in comparison to the pre-pandemic year. The top panel includes all hospitalizations, while the bottom panel stratifies hospitalizations by discharge destination. (absolute values for geometric mean length of discharging stay are depicted in Additional file 8). The *circles* represent observed values unadjusted for potential confounders; the sizes of the circles are proportional to the number of annual discharges. The *dashed line* represents the relative increase in geometric mean length of discharging stay when both avoidable bed days and potential confounders are held to mean values from the pre-pandemic year. In contrast, the *solid line* represents the relative increase in geometric mean length of discharging stay when potential confounders are held to mean values from the pre-pandemic year while avoidable bed days are set to the mean value for the corresponding year of hospital discharge. The *error bars* represent 95% confidence intervals. The relative increase for all 3 pandemic years combined (not shown), holding potential confounders to pre-pandemic levels, was 6.1% (95% ci 5.7 to 6.5%) for all hospitalizations; when grouped by discharge destination the increases were 5.8% (5.3 to 6.3%), 9.3% (8.2 to 10.3%), and 17.2% (16.1 to 18.4%) for discharges to home, to at-home post-acute care, and to post-acute facilities, respectively

Unadjusted lengths of stay, both for the discharging stay and the entire hospitalization, increased across the study period. After holding potential confounders to their pre-pandemic levels, the largest increases were among discharges to post-acute care (Fig. 2, Additional File 8). From the pre-pandemic year to pandemic year 3, for example, adjusted geometric mean length of discharging stay for discharges to at-home post-acute care increased by 12.2% (95% CI, 11.0 to 13.6%) and to facility-based post-acute care by 23.2% (95% CI, 21.6 to 24.9%), while those discharged home without post-acute increased by only 8.9% (95% CI, 8.3 to 9.5%).

**Fig. 3:**
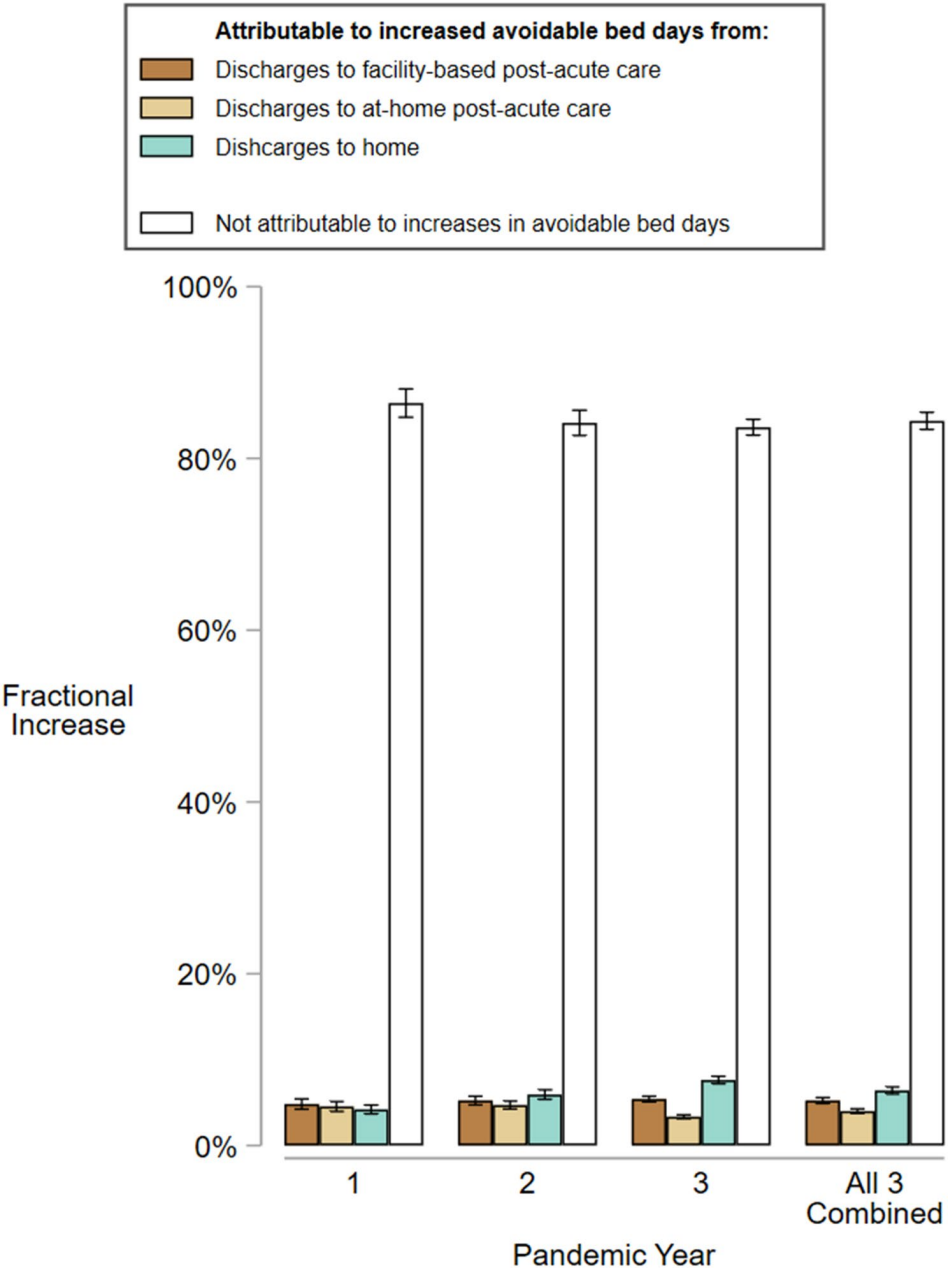
Proportion of overall increase in length of discharging stay attributable to avoidable days. The heights of the bars represent the annual increase in geometric mean length of discharging stay in comparison to the pre-pandemic year for all veterans discharged from acute medicine services. Colored bars represent the fractions of annual increases that are attributable to increases in avoidable bed days accrued before discharge, grouped by eventual discharge destination. Clear bars represent the fractions of annual increases that were not attributable to increases in avoidable bed days. For example, 6.4% (95% CI 6.0 to 6.8%) of the increase in length of discharging stay for all 3 pandemic years combined when compared to the pre-pandemic year is attributable to the increase in avoidable bed days accrued from veterans who were discharged home; 4.0% (3.7 to 4.3%) from veterans discharged to at-home post-acute care, and 5.2% (4.9 to 5.6%) from veterans discharged to facility-based post-acute care; the remaining 84.4% (83.4 to 85.3%) of the increase was not attributable to increases in avoidable bed daysci

The mean number of avoidable days per hospitalization rose from 0.64 in the pre-pandemic year to 1.05 in pandemic year 3 (Table 3). Had this increase in avoidable days not occurred, the adjusted geometric mean length of discharging stay would have increased from 2.91 days (95% CI 2.85 to 2.97 days) during the pre-pandemic year to only 3.13 days (95% CI 3.07 to 3.19 days), instead of 3.18 days (95% CI 3.11 to 3.24 days) during the third pandemic year; the modest difference between these predictions reflects only 16% (95% CI 15 to 17%) of the increase in discharging stay above baseline. This suggests that most of the increase (84%, 95% CI 83 to 85%) was attributable to something other than increases in avoidable days (Fig. 3).

**Table 3:** Avoidable bed days by discharge destination^a^

Year	Hospitalizations	Hospitalizations with at least 1 avoidable bed day during discharging stay	Total Avoidable Bed Days	
	N	No (%)	No.	Mean No per hospitalization
**Discharged to Post-Acute Facility**				
Pre-Pandemic Year	35,307	15,651 (44.3)	68,095	1.93
Pandemic Year 1	24,159	11,327 (46.9)	63,187	2.62
Pandemic Year 2	28,497	14,200 (49.8)	79,953	2.81
Pandemic Year 3	26,623	13,912 (52.3)	92,327	3.47
**Discharged to Home with At-Home Post-Acute Care**				
Pre-Pandemic Year	35,369	6,143 (17.4)	18,596	0.53
Pandemic Year 1	33,992	5,686 (16.7)	20,336	0.60
Pandemic Year 2	38,643	6,733 (17.4)	23,537	0.61
Pandemic Year 3	37,363	6,710 (18.0)	29,000	0.78
**Discharged Home**				
Pre-Pandemic Year	182,109	23,350 (12.8)	74,887	0.41
Pandemic Year 1	136,988	16,910 (12.3)	63,466	0.46
Pandemic Year 2	146,046	18,892 (12.9)	75,975	0.52
Pandemic Year 3	142,935	18,935 (13.3)	95,915	0.67
**All Hospitalizations**				
Pre-Pandemic Year	252,785	45,144 (17.9)	161,578	0.64
Pandemic Year 1	195,139	33,923 (17.4)	146,989	0.75
Pandemic Year 2	213,186	39,825 (18.7)	179,465	0.84
Pandemic Year 3	206,921	39,557 (19.1)	217,242	1.05

^a^ Avoidable bed days were only included in our analysis if they occurred during the discharging stay

## Conclusion

We found that only a small fraction of the increase in adjusted length of discharging stay over 3 pandemic years compared to the pre-pandemic year was attributable to an increase in avoidable days (Fig. 3). Since avoidable days ought to increase alongside discharge delays, this small fraction implies that most of the increase was not related to more discharge delays, suggesting that more time may have been spent on acute care delivery—the period before a patient is medically ready for discharge.

Our findings should be interpreted with 3 important limitations. First, VHA utilization management reviewers, working alone, may have overcounted or undercounted avoidable days. A multidisciplinary team that also included physicians, nurses, and social workers would likely be better equipped to determine whether a patient’s complex social, cognitive, and physical dimensions warrant ongoing hospitalization [5]. Nevertheless, when the purpose of measurement is to assess longitudinal change, stable systematic errors tend to cancel each other out, if that measurement is a reliable one [18]. Because the methods of VHA utilization reviewers are standardized and repeatedly evaluated to maintain high reliability [19], changes in avoidable days (Table 3) were likely to reflect actual change, not mere measurement error. Second, the COVID-19 pandemic may have altered hospital operations and patient behavior. To mitigate this, we excluded veterans diagnosed with COVID-19 and defined our study years relative to the onset of the pandemic. The fact that length of stay had been stable within VHA hospitals in the decade leading up to the pandemic [20], together with the consistent upward trends over 3 consecutive years, suggests that our findings are not merely a transient phenomenon. Third, we did not include some potential confounders in our model because they were not available. For example, nurses’ diagnoses upon hospital admission, such as risk for infection, impaired skin integrity, or disturbed sleep patterns [21], are associated with length of stay [22] and may have increased alongside the complexity of hospitalized patients [23]. While our inclusion of a comorbidity index, along with 15 other potential confounders, did address temporal changes in some of the determinants of length of stay (as evidenced by the difference between the circles and the solid lines in Fig. 2), this was likely incomplete given the vast number of other potential determinants [24].

An ideal measure would be if hospitals adopted a medically-ready-for-discharge indicator, which would allow changes in length of stay to be directly attributed to care delivery or discharge delays [5]. Until then, our findings suggest that targeting discharge delays may have a smaller overall impact on hospital capacity strain than anticipated [25]. Improving acute care delivery, perhaps by bolstering hospital staff and procedural care availability—both of which were diminished by the pandemic at a time when patient complexity was already on the rise [26]—may be more fruitful [26].

## Electronic supplementary material

Below is the link to the electronic supplementary material.

Supplementary Material 1

Supplementary Material 2

Supplementary Material 3

Supplementary Material 4

Supplementary Material 5

Supplementary Material 6

Supplementary Material 7

Supplementary Material 8

Supplementary Material 9

## Data Availability

US Department of Veterans Affairs regulations and our ethics agreements require that the analytic data sets used for this study do not leave the VA firewall without a data use agreement. However, VA data are made freely available to researchers with an approved VA study protocol. For more information visit https://www.virec.research.va.gov.

## Declarations

### Disclaimer

The contents of this publication do not represent the views of the U.S. Department of Veterans Affairs (VA) or the United States Government..

### Author information

BPL has been a practicing adult hospitalist (hospital-based) physician at the White River Junction VAMC, White River Junction, VT, since 2013.
