# Supplementary material for: Do discharge delays explain longer stays at veterans health administration hospitals?
Source: BMC Health Serv Res. 2025 Dec 12;25:1595. doi: 10.1186/s12913-025-13682-w (PMC12699839; doi:10.1186/s12913-025-13682-w)
Supplement: Supplementary file 1 — Supplementary Material 1 [file 12913_2025_13682_MOESM1_ESM.docx]

Additional Files

| **Name (Format)** | **Title** | **Description** |
| --- | --- | --- |
| Additional File 1 (PDF) | Corporate Data Warehouse Retrieval | Administrative data obtained from Inpatient 3.0 Production Domain to form hospitalizations |
| Additional File 2 (PDF) | Specialties | Codes used to define specialties |
| Additional File 3 (PDF) | Validation of Method to Identify Specialty Stays | Comparison of weekly national VHA inpatient medicine/surgery census derived from Bed Management Solution versus Corporate Data Warehouse |
| Additional File 4 (PDF) | Cohort Criteria | Definitions of cohort criteria |
| Additional File 5 (PDF) | Hospitals | List of VHA medical centers involved |
| Additional File 6 (PDF) | Exposures of Interest and Potential Confounders | Definitions of exposures of interest and potential confounders |
| Additional File 7 (PDF) | Quantile Normal Plots of Random Effects | Diagnostic check of the underlying assumptions of our statistical model |
| Additional File 8 (PDF) | Length of Discharging Stay | Yearly values of geometric mean length of discharging stay by discharge destination |
