# Supplementary material for: Do discharge delays explain longer stays at veterans health administration hospitals?
Source: BMC Health Serv Res. 2025 Dec 12;25:1595. doi: 10.1186/s12913-025-13682-w (PMC12699839; doi:10.1186/s12913-025-13682-w)
Supplement: Supplementary file 2 — Supplementary Material 2 [file 12913_2025_13682_MOESM2_ESM.docx]

**Inpatient**

InpatientSID ●

PatientSID & Sta3n

AdmitDateTime

DischargeDateTime

DispositionType

Discharge45SpecialtySID^a^

→ Specialty

AdmitWardLocationSID^a^

→ Sta6a

Discharge45WardLocationSID^a^

→ Sta6a

WHERE DischargeDateTime is on or after 1 March 2019 AND DischargeDateTime is on or before 28 February 2023.

**SpecialtyTransfer**

● InpatientSID

SpecialtyTransferDateTime

MovementDateTime

LosingSpecialtySID^a^

→Specialty

TreatingSpecialtySID^a^

→Specialty

RelatedPhysicalPatientTransferSID^b^

→LosingWardLocationSID

→Sta6a

RelatedPhysicalPatientTransferSID^b^

→GainingWardLocationSID

→Sta6a

WHERE SpecialtyTransferDateTime not null

**Inpatient535Transaction**

● InpatientSID

MovementDateTime

LosingWardLocationSID^a^

→ Sta6a

WHERE MovementDateTime not null

**Inpatient501Transaction**

InpatientSID ●

MovementDateTime

LosingSpecialtySID^a^

→ Specialty

WHERE MovementDateTime not null

Each *box* represents a table within the Inpatient 3.0 CDW Production Domain. The *black arrows* represent linkages between tables, using the inpatient security identifier (SID). ^a^Some specialty and location codes were obtained with direct linkages to dimension (‘dim’) tables in the CDW Work domain. ^b^Other location codes were obtained by first linking to the Patient Transfer table (using the Patient Transfer SID) and then to the dimension tables.
