# Supplementary material for: Do discharge delays explain longer stays at veterans health administration hospitals?
Source: BMC Health Serv Res. 2025 Dec 12;25:1595. doi: 10.1186/s12913-025-13682-w (PMC12699839; doi:10.1186/s12913-025-13682-w)
Supplement: Supplementary file 3 — Supplementary Material 3 [file 12913_2025_13682_MOESM3_ESM.docx]

| **Specialty** | **Codes^b^** |
| --- | --- |
| Observation |  |
| Medicine or Surgery | MEDICAL OBSERVATION; SURGICAL OBSERVATION |
| Not Medicine or Surgery | PSYCHIATRIC OBSERVATION; ED OBSERVATION; SPINAL CORD INJURY OBSERVATION; NEUROLOGY OBSERVATION; REHAB MEDICINE OBSERVATION |
| Acute |  |
| Medicine | GENERAL(ACUTE MEDICINE); CARDIOLOGY; GASTROENTEROLOGY; METABOLIC; HEMATOLOGY/ONCOLOGY; GEM INTERMEDIATE CARE; GEN MEDICINE (ACUTE) PULMONARY, NON-TB; PULMONARY, TUBERCULOSIS; HOSPICE FOR ACUTE CARE; TELEMETRY; INTERMEDIATE MEDICINE; GRECC-MED; DERMATOLOGY |
| Surgery | ANESTHESIOLOGY; CARDIAC SURGERY; GENERAL SURGERY; NEUROSURGERY; THORACIC SURGERY; TRANSPLANTATION; UROLOGY; VASCULAR; OB/GYN; OPHTHALMOLOGY; ORAL SURGERY; ORTHOPEDIC; PERIPHERAL VASCULAR; PLASTIC SURGERY; EAR, NOSE, THROAT (ENT); PODIATRY |
| Mental Health | GEN INTERMEDIATE PSYCH; HIGH INTENSITY GEN PSYCH INPAT; HIGH INTENSITY GEN INPT; SUBSTANCE ABUSE TRMT UNIT; SIPU (SPEC INPT PTSD UNIT); ALCOHOL DEPENDENCE TRMT UNIT; ACUTE PSYCHIATRY (<45 DAYS); GEM PSYCHIATRY; PSYCHIATRY |
| Other | EPILEPSY CENTER; NEUROLOGY; SPINAL CORD INJURY; REHABILITATION MEDICINE; POLYTRAUMA REHAB UNIT |
| Intensive Care | CARDIAC INTENSIVE CARE UNIT; MEDICAL ICU; SURGICAL ICU; CARDIAC STEP DOWN UNIT; CARDIAC-STEP DOWN UNIT; MEDICAL STEP DOWN; SURGICAL STEPDOWN |
| Extended Nursing | NH GEM NURSING HOME CARE; NH HOSPICE; NH LONG STAY DEMENTIA CARE; NH LONG STAY SKILLED NURSING; NH LONG STAY SPINAL CORD INJ; NH LONG-STAY CONTINUING CARE; NH LONG-STAY MH RECOVERY; NH RESPITE CARE (NHCU); NH SHORT STAY DEMENTIA CARE; NH SHORT STAY MAINTENANCE; NH SHORT STAY PSYCHIATRIC CARE; NH SHORT STAY REHABILITATION; NH SHORT STAY RESTORATIVE; NH SHORT STAY SKILLED NURSING; NH SHORT-STAY CONTINUING CARE; NH SHORT-STAY MH RECOVERY; NHCU; STAR I, II & III; PM&R TRANSITIONAL REHAB; BLIND REHAB; SPINAL CORD INJURY LTC CENTER; NH LONG STAY MAINTENANCE CARE; NH LONG STAY PSYCHIATRIC CARE; LONG STAY GRECC-NHCU; SHORT STAY GRECC-NHCU |
| Housing | DOMICILIARY; DOMICILIARY CHV; DOMICILIARY PTSD; DOMICILIARY SUBSTANCE ABUSE; GENERAL CWT/TR; RESPITE CARE (MEDICINE); DOMICILIARY SUBSTANCE USE DO SUBSTANCE ABUSE RESID PROG; PSYCH RESID REHAB PROG; PSYCH RESID REHAB TRMT PROG; PTSD RESIDENTIAL REHAB PROG; DOMICILIARY GENERAL; PTSD RESID REHAB PROG; HOMELESS CWT/TRANS RESID |

^a^Specialties are also known as services or specialty services. ^b^Codes were obtained from fields and tables within the Corporate Data Warehouse Inpatient 3.0 Production Domain: *LosingSpecialtySID* and *TreatingSpecialtySID* from the *SpecialtyTransfer* table, *Discharge45SpecialtySID* from the *Inpatient* table, and *LosingSpecialtySID* from the *Inpatient501Transaction* table.
