# Supplementary material for: Do discharge delays explain longer stays at veterans health administration hospitals?
Source: BMC Health Serv Res. 2025 Dec 12;25:1595. doi: 10.1186/s12913-025-13682-w (PMC12699839; doi:10.1186/s12913-025-13682-w)
Supplement: Supplementary file 4 — Supplementary Material 4 [file 12913_2025_13682_MOESM4_ESM.docx]

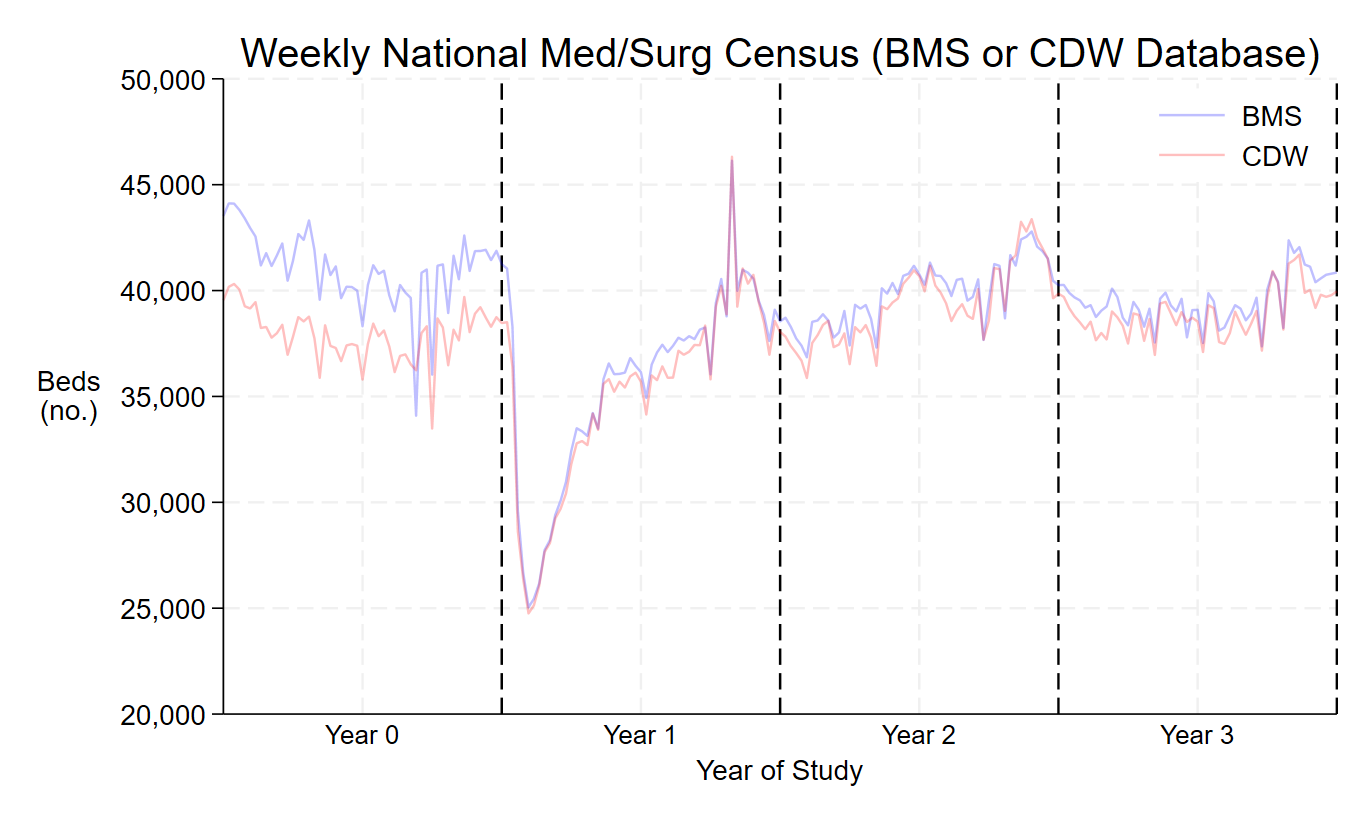


Every day at each VHA medical center a “Bed/Patient Flow Coordinator” enters the number of occupied beds into the Bed Management Solution (BMS) database.^[[1]](#footnote-2)^ We used this database, which is wholly separate from the Corporate Data Warehouse (CDW), to validate how we identified specialty stays using CDW (as described in **Methods**). We compared the BMS-derived (*blue line*) and CDW-derived (*red line*) weekly censuses of combined acute surgery and medicine stays across all 109 VHA medical centers included in our study. (We combined acute medicine and acute surgery stays because BMS does not distinguish between the two specialties.) The totals from both methods were remarkably similar, and the small systematic differences between them are readily explained. The BMS census tends to be higher than the CDW census because the underlying units of measure are different. The CDW census is generated from individual beds, while the BMS census is generated from sets of beds called ward-groups. All beds in a ward-group are counted together. For example, the census for a 5-bed ward-group that is occupied with 1 acute medicine patient, 1 acute surgery patient, and 1 acute mental health patient is 3. The BMS census for acute medicine or surgery stays would include all 3 beds in this ward-group, including the acute mental health patient. Because the units of measure in BMS (ward-groups) are more course than in CDW (beds) the BMS census is predictably higher than the CDW census. How much higher depends upon the granularity of ward-groups. This explains why the differences between the two methods were more extreme in the year leading up to the pandemic (*Year 0*). In response to the COVID-19 pandemic and the need to more reliably track the availability of inpatient beds by type, the BMS database balkanized ward-groups into smaller sets of beds, thereby improving the ability to track certain bed types, such as those with and without respiratory isolation. This systematic change accounts for the improved similarity of census totals after the pandemic (*Year 1* through *Year 3*). Abbreviations: BMS = Bed Management Solution; CDW = Corporate Datawarehouse.

1. Veterans Health Administration. VHA Publications. VHA Directive 1002. https://www.va.gov/vhapublications/index.cfm . Updated November 28, 2017. Accessed April 25, 2025. [↑](#footnote-ref-2)
