# Supplementary material for: Do discharge delays explain longer stays at veterans health administration hospitals?
Source: BMC Health Serv Res. 2025 Dec 12;25:1595. doi: 10.1186/s12913-025-13682-w (PMC12699839; doi:10.1186/s12913-025-13682-w)
Supplement: Supplementary file 5 — Supplementary Material 5 [file 12913_2025_13682_MOESM5_ESM.docx]

This table is designed to be used alongside **Figure 1: Cohort Creation and Data Linkages** from the main paper. Grey-bar headings indicate data sources.

| **Criterion** | **Definition** | | |
| --- | --- | --- | --- |
| **Corporate Data Warehouse** | | | |
| Eligible hospitalizations | **Alive on discharge:** Date of death from VHA vital status file was missing, and the discharge disposition field from the *Inpatient* table (*Inpatient 3.0* CDW Production Domain) was not equal to “DEATH”.  Sohn et al^[[1]](#footnote-2)^ found that, in comparison to the National Death Index (NDI), the VHA vital status file had a sensitivity and specificity of 98.3% and 99.8%, respectively. In addition, the date of death from the VHA vital status death date exactly matched the NDI date of death 98% of the time.  **Discharged from VHA medical center with an emergency department**: The 109 VHA medical centers with emergency departments in the continental United Status are listed in **Additional File 5**. Medical centers with emergency departments are more representative of typical US hospitals; data from the National Emergency Department Inventory suggests that 90% of US hospitals have emergency departments.^[[2]](#footnote-3)^  **Discharged from VHA medical center in the Continental United States**: Medical centers outside the Continental United States are importantly distinct in terms of post-acute care needs and availability.^[[3]](#footnote-4)^ | | |
| Discharged from Acute Medicine Service | The *inpatientsid* field from the *Inpatient* table (*Inpatient 3.0* CDW Production Domain) represents a ‘bedded stay,’ not a hospitalization. A bedded stay is when a patient is provided a bed in a VHA facility. Each bedded stay is comprised of one or more specialty stays. (**Additional File 2** lists the grouping of specialties into 9 categories.) After removing 2 categories of specialty stays (Nursing and Housing), we joined continuous specialty stays together, using the methods of Vincent et al,^[[4]](#footnote-5)^ to create a dataset of hospitalizations with timestamped movements between specialty and location.  We identified hospitalizations where patients signed out against medical advice as the *FacilityMovementType* field (*Inpatient 3.0* CDW Production Domain) equal to “irregular” for the last bedded stay within a hospitalization. | | |
| Not diagnosed with COVID-19 | Neither the primary ICD10-CM code from *PrincipalDiagnosisICD10SID* field from the *Inpatient* table nor any secondary code from the *InpatientDiagnosisSID* field from the *InpatientDiagnosis* was equal to U07.1, U07.2, or J12.82 for any bedded stay in a hospitalization. | | |
| **Geriatrics & Extended Care Data Analysis Center (GECDAC) Residential History File** | | | |
| Not enrolled in hospice. | Patients were enrolled in hospice if any 1 of the 9 *HEE_*TYPE fields (*HEE_TYPE1* through HEE_TYPE9) from the EFB Residential History File^[[5]](#footnote-6)^ equaled any of the following codes within 3 days of hospital admission or within 7 days of hospital discharge. | | |
|  | 4. HSPC CNH VA CDS  4. HSPC CNH VA FEE  4. HSPC MDCR COMMUNITY  4. HSPC MDCR COMMUNITY NH  4. HSPC MDCR INPAT  4. HSPC MDCR INPAT NH  4. HSPC VA ACUTE  4. HSPC VA ACUTE MDS  4. HSPC VA CDS COMMUNITY  4. HSPC VA CDS COMMUNITY MDS  4. HSPC VA CDS INPAT  4. HSPC VA CDS INPAT MDS  4. HSPC VA CLC | | 4. HSPC VA CLC MDS  4. HSPC VA CLC TRTSP  4. HSPC VA CLC TRTSP MDS  4. HSPC VA FEE HSPC/PC  4. HSPC VA FEE HSPC/PC MDS  4. HSPC VA FEE INPAT  4. HSPC VA MILL  4. HSPC VA MILL CLC  4. HSPC VA MILL CLC MDS  4. HSPC VA MILL INP  4. HSPC VA MILL INP ICU  4. HSPC VA MILL INP MDS  4. HSPC VA MILL MDS |
| Discharged home or to a post-acute facility | Patients were discharged home or to a post-acute care facility if they were 1) not transferred for ongoing acute care to a non-VHA facility (including a non-VHA a long-term care hospital [LTCH]) or 2) not discharged to a VHA domiciliary.  Patients were transferred to a non-VHA facility for ongoing acute care if the *HEE_TYPE1* field from EFB Residential History File^4^ was equal to any of the following within 1 day of hospital discharge: | | |
|  | *Non-VHA Hospital Acute Care* | | |
|  | 1. INP MCAID TAF IP  1. INP MCAID TAF IP ICU  1. INP MCO  1. INP MCO ICU  1. INP MDCR ACUTE  1. INP MDCR ACUTE ICU  1. INP MDCR CAH  1. INP MDCR CAH ICU  1. INP MDCR LTCH  1. INP MDCR LTCH ICU  1. INP MDCR OTHER  1. INP MDCR OTHER ICU  1. INP MDCR PSYC  1. INP MDCR PSYC ICU  1. INP MDCR REHAB  1. INP MDCR REHAB ICU  1. INP MPI ACUTE  1. INP MPI ACUTE ICU  1. INP MPI CAH  1. INP MPI CAH ICU  1. INP MPI LTCH  1. INP MPI LTCH ICU | 1. INP MPI OTHER  1. INP MPI OTHER ICU  1. INP MPI PSYC  1. INP MPI REHAB  1. INP MPI PSYC ICU  1. INP MPI REHAB ICU  1. INP VA CDS ACUTE  1. INP VA CDS ACUTE ICU  1. INP VA CDS CAH  1. INP VA CDS CAH ICU  1. INP VA CDS LTCH  1. INP VA CDS LTCH ICU  1. INP VA CDS MULTI  1. INP VA CDS MULTI ICU  1. INP VA CDS OTHER  1. INP VA CDS OTHER ICU  1. INP VA CDS PSYC  1. INP VA CDS PSYC ICU  1. INP VA CDS REHAB  1. INP VA CDS REHAB ICU  1. INP VA FEE | |
|  | Patients were discharged to a domiciliary if the *HEE_TYPE1* field from EFB Residential History File was equal to “14.DOM VA” within 3 days of hospital discharge. | | |

1. Sohn MW, Arnold N, Maynard C, Hynes DM. Accuracy and completeness of mortality data in the Department of Veterans Affairs. *Popul Health Metr* 2006;4:2. [↑](#footnote-ref-2)
2. Boggs KM, Augustine JJ, Sullivan AF, Espinola JA, Camargo CA. Changes in the Number of United States Emergency Departments and Their Annual Visit Volumes Since 2001. *Ann Emerg Med* 2023;82(6):760–762. [↑](#footnote-ref-3)
3. Rivera-Hernandez M, Matos-Moreno A, Ferdows NB, Kumar A. Posthospital nursing home utilization and quality indicators among Medicare beneficiaries in Puerto Rico: comparison with the United States. *J Am Med Dir Assoc* 2021;22(3):712–716. [↑](#footnote-ref-4)
4. Vincent BM, Wiitala WL, Burns JA, Iwashyna TJ, Prescott HC. Using veterans affairs Corporate Data Warehouse to identify 30-day hospital readmissions. *Health Serv Outcomes Res Method* 2018;18:143–154. [↑](#footnote-ref-5)
5. Intrator O, Li J, Gillespie SM, Levy C, Davis D, Edes T, Kinosian B, Karuza J. Benchmarking site of death and hospice use: a case study of veterans cared by Department of Veterans Affairs home-based primary care. *Med Care* 2020;58(9):805–14. [↑](#footnote-ref-6)
