# Supplementary material for: Do discharge delays explain longer stays at veterans health administration hospitals?
Source: BMC Health Serv Res. 2025 Dec 12;25:1595. doi: 10.1186/s12913-025-13682-w (PMC12699839; doi:10.1186/s12913-025-13682-w)
Supplement: Supplementary file 6 — Supplementary Material 6 [file 12913_2025_13682_MOESM6_ESM.docx]

| **Hospital (VHA identifier, State)** | **CDC Region** | **Rurality** | **CLC on Campus** |
| --- | --- | --- | --- |
| IOWA CITY VA MEDICAL CENTER (636A8, IA) | MW | U | NO |
| DES MOINES - VA CENTRAL IOWA HEALTH CARE SYSTEM (636A6, IA) | MW | U | YES |
| MARION, ILLINOIS VAMC (657A5, IL) | MW | U | YES |
| JESSE BROWN VA MEDICAL CENTER (537, IL) | MW | U | YES |
| NORTH CHICAGO VA MEDICAL CENTER (556, IL) | MW | U | YES |
| HINES (578, IL) | MW | U | YES |
| RICHARD L. ROUDEBUSH-INDIANAPOLIS VA MEDICAL CENTER (583, IN) | MW | U | NO |
| VA NORTHERN INDIANA HEALTH CARE SYSTEM (610A4, IN) | MW | U | NO |
| ROBERT J. DOLE VAMC (589A7, KS) | MW | U | YES |
| VA EASTERN KANSAS-DWIGHT D. EISENHOWER VAMC (589A6, KS) | MW | R | YES |
| VA EASTERN KANSAS HEALTH CARE SYSTEM - TOPEKA (589A5, KS) | MW | U | YES |
| JOHN D. DINGELL VA MEDICAL CENTER (553, MI) | MW | U | YES |
| VA ANN ARBOR HEALTHCARE SYSTEM (506, MI) | MW | U | YES |
| MINNEAPOLIS VA MEDICAL CENTER (618, MN) | MW | U | YES |
| ST. LOUIS VA MEDICAL CENTER - JOHN COCHRAN DIVISION (657, MO) | MW | U | NO |
| HARRY S. TRUMAN MEMORIAL (589A4, MO) | MW | U | YES |
| KANSAS CITY VA MEDICAL CENTER (589, MO) | MW | U | NO |
| FARGO VA HEALTHCARE SYSTEM (437, ND) | MW | U | YES |
| OMAHA-VA NEBRASKA-WESTERN IOWA HEALTH CARE SYSTEM (636, NE) | MW | U | NO |
| DAYTON VA MEDICAL CENTER (552, OH) | MW | U | YES |
| CINCINNATI VA MEDICAL CENTER (539, OH) | MW | U | YES |
| LOUIS STOKES VA MEDICAL CENTER (541, OH) | MW | U | YES |
| SIOUX FALLS VA HEALTH CARE SYSTEM (438, SD) | MW | U | YES |
| VA BLACK HILLS HEALTH CARE SYSTEM-FORT MEADE CAMPUS (568, SD) | MW | R | YES |
| CLEMENT J. ZABLOCKI VETERANS AFFAIRS MEDICAL CENTER (695, WI) | MW | U | YES |
| WILLIAM S. MIDDLETON MEMORIAL VETERANS HOSPITAL (607, WI) | MW | U | YES |
| VA CONNECTICUT HEALTHCARE SYSTEM WEST HAVEN CAMPUS (689, CT) | NE | U | YES |
| VA BOSTON HEALTHCARE SYSTEM, WEST ROXBURY CAMPUS (523A4, MA) | NE | U | NO |
| VA ME HCS (402, ME) | NE | R | YES |
| VA NEW JERSEY HEALTH CARE SYSTEM-EAST ORANGE (561, NJ) | NE | U | NO |
| SYRACUSE VA MEDICAL CENTER (528A7, NY) | NE | U | YES |
| NORTHPORT VA MEDICAL CENTER (632, NY) | NE | U | YES |
| ALBANY VA MEDICAL CENTER: SAMUEL S. STRATTON (528A8, NY) | NE | U | YES |
| VA WESTERN NEW YORK HEALTHCARE SYSTEM AT BUFFALO (528, NY) | NE | U | YES |
| VA NY HARBOR HEALTHCARE SYSTEM-BROOKLYN (630A4, NY) | NE | U | NO |
| NEW YORK CAMPUS OF VA NY HARBOR HEALTHCARE SYSTEM (630, NY) | NE | U | NO |
| JAMES J. PETERS VA MEDICAL CENTER (BRONX, NY) (526, NY) | NE | U | YES |
| LEBANON VA MEDICAL CENTER (595, PA) | NE | U | YES |
| WILKES-BARRE VA MEDICAL CENTER (693, PA) | NE | U | YES |
| VA PITTSBURGH HEALTHCARE SYSTEM, UNIVERSITY DR DIV (646, PA) | NE | U | NO |
| CORPORAL MICHAEL J. CRESCENZ VAMC (PHILADELPHIA, PA) (642, PA) | NE | U | YES |
| PROVIDENCE VA MEDICAL CENTER (650, RI) | NE | U | NO |
| WHITE RIVER JUNCTION VA MEDICAL CENTER (405, VT) | NE | R | NO |
| BIRMINGHAM VA MEDICAL CENTER (521, AL) | S | U | NO |
| CENTRAL ALABAMA VETERANS HEALTH CARE SYSTEM (619, AL) | S | U | NO |
| VETERANS HEALTH CARE SYSTEM OF THE OZARKS (564, AR) | S | U | NO |
| JOHN L. MCCLELLAN MEMORIAL VETERANS HOSPITAL (598, AR) | S | U | NO |
| WASHINGTON DC VA MEDICAL CENTER (688, DC) | S | U | YES |
| WILMINGTON VA MEDICAL CENTER (460, DE) | S | U | YES |
| BAY PINES VA HEALTHCARE SYSTEM (516, FL) | S | U | YES |
| JAMES A. HALEY VETERANS&#39; HOSPITAL (673, FL) | S | U | YES |
| GAINESVILLE VAMC (573, FL) | S | U | YES |
| LAKE CITY (573A4, FL) | S | R | YES |
| MIAMI VA HEALTHCARE SYSTEM (546, FL) | S | U | YES |
| ORLANDO VA MEDICAL CENTER (675, FL) | S | U | YES |
| WEST PALM BEACH VAMC (548, FL) | S | U | YES |
| ATLANTA VA MEDICAL CENTER (508, GA) | S | U | YES |
| CHARLIE NORWOOD VA MEDICAL CENTER (509, GA) | S | U | NO |
| LEXINGTON VA HEALTH CARE SYSTEM (596A4, KY) | S | U | NO |
| LOUISVILLE VA MEDICAL CENTER (603, KY) | S | U | NO |
| SOUTHEAST LOUISIANA VETERANS HEALTHCARE SYSTEM (629, LA) | S | U | YES |
| OVERTON BROOKS VA MEDICAL CENTER (667, LA) | S | U | NO |
| VA MARYLAND HEALTH CARE SYSTEM (512, MD) | S | U | NO |
| G.V. (SONNY) MONTGOMERY VA MEDICAL CENTER (586, MS) | S | U | YES |
| VA GULF COAST VETERANS HEALTH CARE SYSTEM (520, MS) | S | U | YES |
| DURHAM VAMC (558, NC) | S | U | YES |
| ASHEVILLE VA MEDICAL CENTER (637, NC) | S | U | YES |
| SALISBURY - W.G. (BILL) HEFNER VA MEDICAL CENTER (659, NC) | S | U | YES |
| JACK C. MONTGOMERY VAMC (623, OK) | S | R | NO |
| OKLAHOMA CITY VA HCS (635, OK) | S | U | YES |
| RALPH H. JOHNSON VA MEDICAL CENTER (534, SC) | S | U | YES |
| WM. JENNINGS BRYAN DORN VA MEDICAL CENTER (544, SC) | S | U | YES |
| TENNESSEE VALLEY HEALTHCARE SYSTEM-NASHVILLE (626, TN) | S | U | NO |
| MOUNTAIN HOME VA MEDICAL CENTER (621, TN) | S | U | YES |
| MEMPHIS VA MEDICAL CENTER (614, TN) | S | U | NO |
| MICHAEL E. DEBAKEY VA MEDICAL CENTER (580, TX) | S | U | YES |
| SOUTH TEXAS VETERANS HEALTH CARE SYSTEM (671, TX) | S | U | YES |
| AMARILLO VA HEALTH CARE SYSTEM (504, TX) | S | U | YES |
| CENTRAL TEXAS VETERANS HEALTH CARE SYSTEM (674, TX) | S | U | YES |
| VA NORTH TEXAS HEALTH CARE SYSTEM: DALLAS (549, TX) | S | U | YES |
| HUNTER HOLMES MCGUIRE VA MEDICAL CENTER (652, VA) | S | U | YES |
| SALEM VA MEDICAL CENTER (658, VA) | S | U | YES |
| HAMPTON VA MEDICAL CENTER (590, VA) | S | U | YES |
| BECKLEY VA MEDICAL CENTER (517, WV) | S | U | YES |
| CLARKSBURG - LOUIS A. JOHNSON VA MEDICAL CENTER (540, WV) | S | R | YES |
| HUNTINGTON VA MEDICAL CENTER (581, WV) | S | U | NO |
| MARTINSBURG VA MEDICAL CENTER (613, WV) | S | U | YES |
| SOUTHERN ARIZONA VA HEALTH CARE SYSTEM (678, AZ) | W | U | YES |
| NORTHERN ARIZONA VA HEALTH CARE SYSTEM (649, AZ) | W | U | YES |
| PHOENIX VA HEALTH CARE SYSTEM (644, AZ) | W | U | YES |
| VA PALO ALTO HEALTH CARE SYSTEM (640, CA) | W | U | YES |
| VA GREATER LOS ANGELES HEALTHCARE SYSTEM (691, CA) | W | U | YES |
| VA LONG BEACH HEALTHCARE SYSTEM (600, CA) | W | U | YES |
| VA CENTRAL CALIFORNIA HEALTH CARE SYSTEM (570, CA) | W | U | YES |
| VA SAN DIEGO HEALTHCARE SYSTEM (664, CA) | W | U | YES |
| VA LOMA LINDA HEALTHCARE SYSTEM (605, CA) | W | U | YES |
| VA NORTHERN CALIFORNIA HEALTH CARE SYSTEM (612A4, CA) | W | U | NO |
| SAN FRANCISCO VA MEDICAL CENTER (662, CA) | W | U | YES |
| VA EASTERN COLORADO HEALTH CARE SYSTEM (ECHCS) (554, CO) | W | U | NO |
| GRAND JUNCTION VA MEDICAL CENTER (575, CO) | W | U | YES |
| BOISE VA MEDICAL CENTER (531, ID) | W | U | YES |
| VA MONTANA (436, MT) | W | R | NO |
| RAYMOND G. MURPHY VAMC (501, NM) | W | U | YES |
| VA SIERRA NEVADA HEALTH CARE SYSTEM (654, NV) | W | U | YES |
| VA SOUTHERN NEVADA HEALTHCARE SYSTEM (VASNHS) (593, NV) | W | U | NO |
| PORTLAND VA MEDICAL CENTER (648, OR) | W | U | NO |
| VA SALT LAKE CITY HEALTH CARE SYSTEM (660, UT) | W | U | NO |
| VA PUGET SOUND HEALTH CARE SYSTEM (663, WA) | W | U | YES |
| CHEYENNE VA MEDICAL (442, WY) | W | U | YES |

Abbreviations: CDC = Centers for Disease Control and Prevention; CLC = Community Living Center; MW = Midwest; R = Rural; S = South; U = Urban; W = West. ^a^All VHA Medical Centers were in the continental United States with an emergency department throughout the study period.
