# Supplementary material for: Do discharge delays explain longer stays at veterans health administration hospitals?
Source: BMC Health Serv Res. 2025 Dec 12;25:1595. doi: 10.1186/s12913-025-13682-w (PMC12699839; doi:10.1186/s12913-025-13682-w)
Supplement: Supplementary file 7 — Supplementary Material 7 [file 12913_2025_13682_MOESM7_ESM.docx]

| **Exposure** | **Definition** | | | |
| --- | --- | --- | --- | --- |
| Year of discharge | Year of discharge was determined by the date of hospital discharge:  *Pre-Pandemic Year*: 1 March 2019 through 29 February 2020  *Pandemic Year 1*: 1 March 2020 through 28 February 2021  *Pandemic Year 2*: 1 March 2021 through 28 February 2022  *Pandemic Year 3*: 1 March 2022 through 28 February 2023  Year of discharge was treated as a categorical variable in our statistical models as one of 4 categories: the pre-pandemic year and pandemic years 1 through 3. | | | |
| Discharge destination | **Home**: Patients were discharged *to home* if they did not meet either criterion below (*Home-based post-acute care* or *Facility-based post-acute care*). | | | |
|  | **Home-based post-acute care**: Patients were discharged *to home with post-acute care* if any 1 of the 9 *HEE_*TYPE fields (*HEE_TYPE1* through HEE_TYPE9) from the EFB Residential History File^[[1]](#footnote-2)^ equaled any of the following codes within 7 days of hospital discharge: | | | |
|  | 10. HHA MCO  10. HHA MDCR  10. PCS VA CDS GEC HM/HHA  10. PCS VA CDS GEC PSHC  10. PCS VA CDS GEC RESPITE  10. PCS VA FEE GEC HM/HHA | 10. PCS VA FEE GEC HM/HHA RESPITE  10. PCS VA FEE GEC NONNURSING  10. PCS VA FEE GEC PSHC  10. PCS VA PIT GEC HM/HHA  10. PCS VA PIT GEC PSHC  10. PCS VA PIT GEC RESPITE | | |
|  | **Facility-based post-acute care**: Patients discharged *to a post-acute facility* if the *HEE_TYPE1* field from EFB Residential History File^1^ was equal to any of the following within 3 days of hospital discharge: | | | |
|  | *Skilled Nursing Facility* | | | |
|  | 3. SNF MCO  3. SNF MDCR HB PAID  3. SNF MDCR HB UNPAID  3. SNF MDCR OTHER PAID  3. SNF MDCR OTHER UNPAID | | 3. SNF MDCR SWING PAID  3. SNF MDCR SWING UNPAID  3. SNF MDCR UNKN PAID  3. SNF MDCR UNKN UNPAID | |
|  | *Contract Nursing Home* | | | |
|  | 6. CNH VA CDS  6. CNH VA CDS IN CAH  6. CNH VA CDS REHAB  6. CNH VA CDS REHAB SWING IN CAH  6. CNH VA CDS RESPITE  6. CNH VA CDS RESPITE IN CAH  6. CNH VA CDS SHORTSTAY REHAB  6. CNH VA CDS SWING | | | 6. CNH VA CDS SWING IN CAH  6. CNH VA FEE  6. CNH VA FEE RESPITE  8. MFH BEREAVEMENT  8. MFH MASTERFILE  11. MDS NH  15. NH MCAID TAF LT |
|  | *Community Living Center (VHA Nursing Home)* | | | |
|  | 5. CLC  5. CLC ASIH  5. CLC AUTH ABSENCE  5. CLC GEM  5. CLC LS CONTINUING  5. CLC LS DEMENTIA  5. CLC LS MH RECOVERY  5. CLC LS SCI  5. CLC LS SKILLED  5. CLC MILL  5. CLC MILL GEM SS  5. CLC MILL LS CONTINUING | | 5. CLC MILL LS DEMENTIA  5. CLC MILL RESPITE  5. CLC MILL SS CONTINUING  5. CLC MILL SS REHAB  5. CLC NHCU  5. CLC REHAB  5. CLC RESPITE  5. CLC RESPITE TRTSP  5. CLC SCIURY LTC CENTER  5. CLC SS CONTINUING  5. CLC SS MH RECOVERY  5. CLC SS SKILLED | |
|  | *State Veterans Home* | | | |
|  | 9. SVH VA | | 11. MDS SVH | |
| Avoidable bed days | Reviews from the National Utilization Management Integration (NUMI) database that were conducted during a patient’s final acute medicine stay were included. These reviews were concurrent because they happened during hospital treatment. (In contrast, prospective and retrospective utilization reviews happen before or after hospital treatment.)  A bed day was classified as ‘avoidable’ if a reviewer determined that 1) the patient no longer met continued stay criteria for acute care and 2) the recommended level of care was at a lower level of acuity than acute inpatient care, such as at home or in a skilled nursing facility. | | | |

**Potential Confounders**

| **Confounder** | **Definition** | |
| --- | --- | --- |
|  | **Hospital-Level** | |
| Centers for Disease Control region | We used the regional geographic groupings defined by the Centers for Disease Control.^[[2]](#footnote-3)^ See **Additional File 5** for classification by medical center. | |
| Rural | We used the VAST system to determine the rurality classification of VHA Medical Centers.^[[3]](#footnote-4)^ See **Additional File 5** for classification by medical center. | |
| Community Living Center on campus | Community Living Centers (CLCs) are VHA nursing homes. Only some VHA Medical Centers have CLCs on campus. We used the VHA Site Tracking (VAST) system to determine if VHA Medical Centers had a Community Living Center (CLC) on campus.^[[4]](#footnote-5)^ See **Additional File 5** for classification by medical center. | |
|  | **Patient-Level** | |
| Sex | The *Gender* field from VHA CDW Patient 3.0 Domain. | |
| Race | We combined race data from two sources: the VHA CDW Patient 3.0 Domain (*Race* and *LegacyRace* fields) and the Vital Status File (*CMS_RACE_LABEL* field). When a veteran’s race data varied, we used the most assigned race (the mode) from all pooled data across that veteran.  We then generated three categories: 1) White, 2) Black or African American, 3) other, and 4) unknown or missing. The ‘other’ race category included American Indian, Alaska Native, Asian, Native Hawaiian, and other Pacific Islander. | |
| Hispanic | We used the *Ethnicity* field from VHA CDW Patient 3.0 Domain and generated two categories: 1) Hispanic, and 2) not Hispanic, unknown ethnicity, or missing ethnicity. | |
|  | **Hospitalization-Level** | |
| Age at discharge | The time difference between hospital discharge date and date of birth. | |
| Elixhauser Comorbidity Index for in-hospital mortality | The index is based on the prevalence of 38 individual comorbidity measures from ICD-10-CM diagnosis codes during a two-year look-back from the date of hospital discharge; we included current hospitalizations in the look-back to reflect illness at the time of discharge.  We used the Elixhauser Comorbitidy software provided by the Healthcare Cost and Utilization Project.^[[5]](#footnote-6)^ The underlying methodology of the ICD-10-CM version was similar to the ICD-9-CM version but used more recent 2018 data.^[[6]](#footnote-7)^ | |
| Pre-hospital care | **Services at home**: Patients received pre-hospital services at home if any 1 of the 9 *HEE_*TYPE fields (*HEE_TYPE1* through HEE_TYPE9) from the EFB Residential History File^[[7]](#footnote-8)^ equaled any of the *Home-Based Post-Acute Care* codes listed in the *Discharge destination* criterion (see *Exposures of Interest* above) within 3 days of hospital admission. | |
|  | **Post-Acute facility**: Patients received pre-hospital care at a post-acute facility if the *HEE_TYPE1* field from EFB Residential History File7 was equal to any of the *Skilled Nursing Facility*, *Contract Nursing Home*, *Community Living Center*, or *State Veterans Home* codes in the *Discharge destination* criterion (see *Exposures of Interest* above) within 3 days of hospital admission. | |
|  | **None:** Patients received no pre-hospital care if they did not meet either criterion above (S*ervices at home* or *post-acute facility*). | |
| Transfer from a non-VHA hospital | Patients were transferred from a non-VHA hospital if the *HEE_TYPE1* field from EFB Residential History File was timestamped within 1 day of hospital admission and equal to any of the *Non-VHA Emergency Department or Observation Status* codes below or any of the *Non-VHA Hospital Acute Care* codes listed as criterion for *Discharged home to a post-acute care facility* (**eTable 1**). | |
|  | *Non-VHA Emergency Department or Observation Status* | |
|  | 7. ED ADMISSION MCAID TAF 0450  7. ED ADMISSION MDCR  7. ED ADMISSION VA CDS  7. ED MCAID TAF  7. ED MCO  7. ED MDCR  7. ED VA CDS  7. ED VA FEE  7. ED 0450 INP MCO  7. ED 0451 INP MCO  7. ED 0456 INP MCO | 7. ED 0459 INP MCO  7. ED 0981 INP MCO  7. ED ADMISSION MCAID TAF 0451  7. ED ADMISSION MCAID TAF 0456  7. ED ADMISSION MCAID TAF 0459  7. ED ADMISSION MCAID TAF 0981  7. OBS/TRT MCAID TAF  7. OBS/TRT MCO  7. OBS/TRT MDCR  7. OBS/TRT VA CDS  7. OBS/TRT ADMISSION VA CDS |
| Observation status on admission | Patients were admitted as observation status if their first specialty stay of a hospitalization was *Observation Status Med-Surg* or *Observation Status not Med-Surg* (**Additional File 2**). | |
| Other specialty stays | Patients had a previous stay on *Acute Mental Health*, *Acute Surgery*, or *Intensive Care* if they had any corresponding specialty stay listed in **Additional File 2** throughout their hospitalization. | |
| Post-acute facility type | The discharge destination for patients who were discharged to a post-acute facility within 3 days of hospital discharge was further classified based on the type of facility. Patients were discharged to a *Skilled Nursing Facility*, *Contract Nursing Home*, *Community Living Center*, or *State Veterans Home* if the *HEE_TYPE1* field from EFB Residential History File^[[8]](#footnote-9)^ was equal to the corresponding codes listed in the *Discharge destination* criterion (**Additional File 4**). | |
| Same pre- and post-hospital care | Using *Pre-Hospital Care* and *Post-Acute Facility Type* criteria above, as well as the *Discharge Destination* criterion in **Additional File 4**, patients were classified into 1 of 4 categories based on a comparison of the type of pre- and post-hospital care received:  1 Admitted to and from home without services  2 Admitted to and from home with services  3 Admitted to and from the same type of post-acute facility  4 Discharged to a different type of care than received pre-hospital | |

Abbreviations: ICD-9-CM = International Classification of Diseases, Ninth Revision, Clinical Modification; ICD-10-CM = International Classification of Diseases, Tenth Revision, Clinical Modification. *Grey-bar* headings indicate nesting level for our mixed-effects model (see **Methods** in main paper). Hospitalizations are nested within both patients and hospitals because many patients have more than one hospitalization and not all individual patient’s hospitalizations occurred at one hospital.

1. Intrator O, Li J, Gillespie SM, Levy C, Davis D, Edes T, Kinosian B, Karuza J. Benchmarking site of death and hospice use: a case study of veterans cared by Department of Veterans Affairs home-based primary care. *Med Care* 2020;58(9):805–14. [↑](#footnote-ref-2)
2. Centers f Centers for Disease Control. National Center for Health Statistics. https://www.cdc.gov/nchs/hus/sources-definitions/geographic-region.htm. Accessed September 10, 2024. [↑](#footnote-ref-3)
3. VHA Support Service Center. VHA Site Tracking (VAST) Snapshot Site Classifcation.. https://vssc.med.va.gov/VSSCMainApp/products (only available through the VHA Intranet). Accessed September 10, 2024. [↑](#footnote-ref-4)
4. VHA Support Service Center. VHA Site Tracking (VAST) Co-Located Program Report. https://vssc.med.va.gov/VSSCMainApp/products (only available through the VHA Intranet). Accessed September 10, 2024. [↑](#footnote-ref-5)
5. Agency for Healthcare Research and Quality. Healthcare Cost & Utilization Project: User support website. Updated January 24, 2025. Accessed on March 1, 2025. https://hcup-us.ahrq.gov/toolssoftware/comorbidityicd10/comorbidity_icd10.jsp [↑](#footnote-ref-6)
6. Moore BJ, White S, Washington R, Coenen N, Elixhauser A. Identifying Increased Risk of Readmission and In-hospital Mortality Using Hospital Administrative Data: The AHRQ Elixhauser Comorbidity Index. *Med Care* 2017l;55(7):698–705. [↑](#footnote-ref-7)
7. Intrator et al. [↑](#footnote-ref-8)
8. Intrator et al. [↑](#footnote-ref-9)
