# Supplementary material for: Do discharge delays explain longer stays at veterans health administration hospitals?
Source: BMC Health Serv Res. 2025 Dec 12;25:1595. doi: 10.1186/s12913-025-13682-w (PMC12699839; doi:10.1186/s12913-025-13682-w)
Supplement: Supplementary file 8 — Supplementary Material 8 [file 12913_2025_13682_MOESM8_ESM.docx]

Panel A


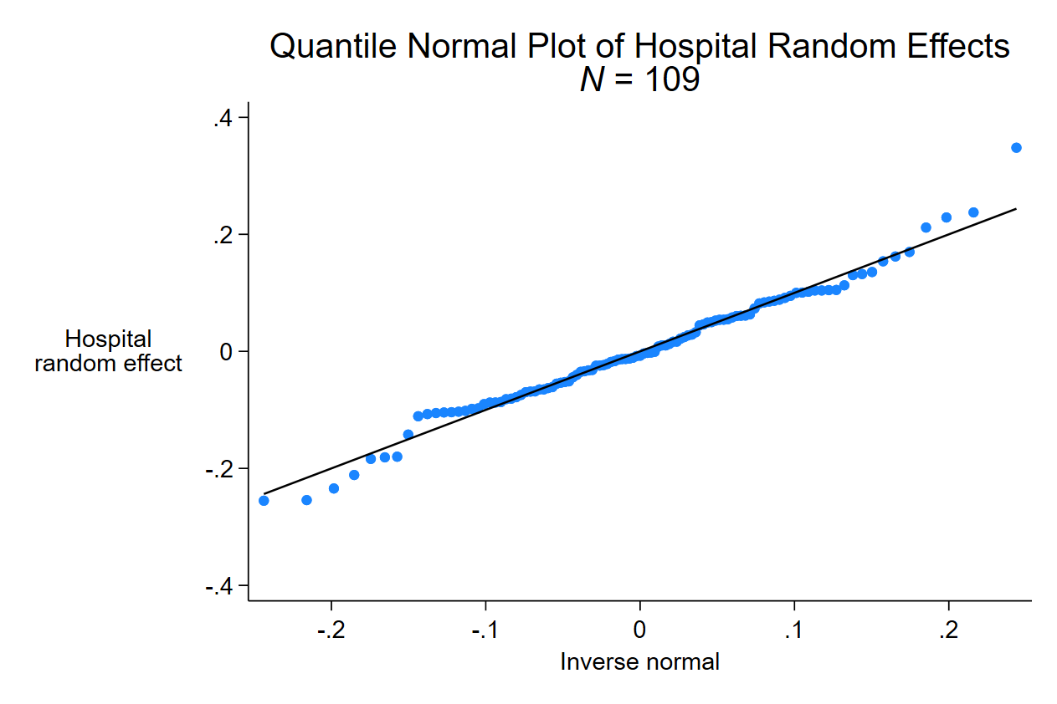


Panel B


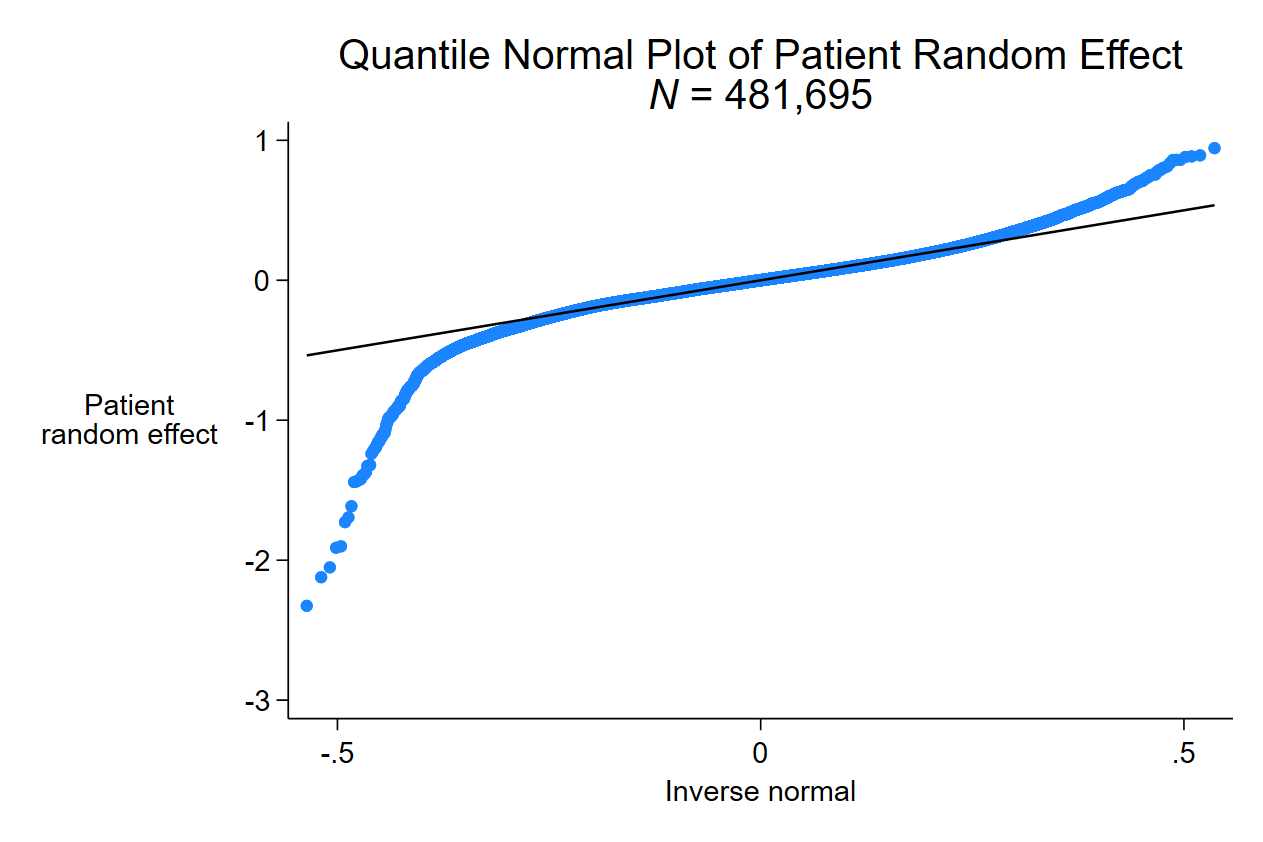


As diagnostic checks on the underlying assumptions of our statistical model, we first used empirical bayes prediction to generate estimates of our random effects. We then used quantile normal plots to assess the assumption that the random effects were normally distributed. The quantile normal plot of 109 VHA hospital random effects follows the *black line* (**Panel A**), suggesting adherence to the normality assumption. In contrast, the quantile normal plot of 481,695 patient random effects veered systematically from the *black line* (**Panel B**), suggesting outliers both below and above the mean patient random effect. In response, we re-ran our model after removing 5% of patients with the largest absolute values of random effects. The model parameters and standard errors did not change substantially, as predicted by statistical theory.^[[1]](#footnote-2)^ We therefore kept these outliers in our final model.

1. Rabe-Hesketh S, Skrondal A. Multilevel and longitudinal modeling using Stata. Volume 1: Continuous responses, 4rd ed. College Station, Texas: Stata Press;2022; p 108, 116–119. [↑](#footnote-ref-2)
