# Supplementary figures and images for: Do discharge delays explain longer stays at veterans health administration hospitals?
Source: BMC Health Serv Res. 2025 Dec 12;25:1595. doi: 10.1186/s12913-025-13682-w (PMC12699839; doi:10.1186/s12913-025-13682-w)

**
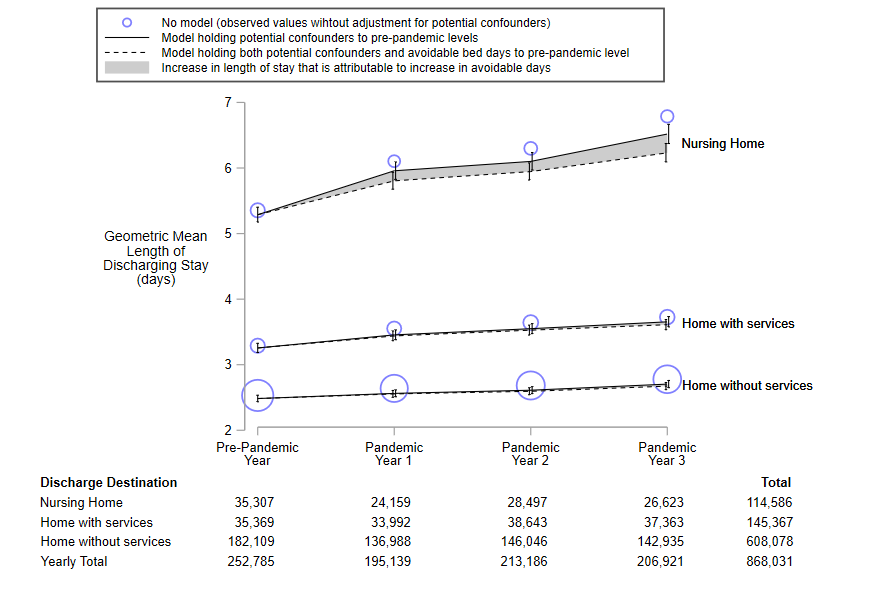
**

The area of the *blue circles* is proportional to the number of annual discharges.

Supplement: Supplementary file 9 — Supplementary Material 9 [file 12913_2025_13682_MOESM9_ESM.docx]
